# Extended Endarterectomy Across the Inguinal Ligament Using Inguinal-Lifting in the Surgery for Critical Limb Ischemia

**DOI:** 10.3400/avd.cr.25-00122

**Published:** 2026-01-20

**Authors:** Anna Tsuji, Shun-Ichiro Sakamoto, Motohiro Maeda, Tomohiro Murata, Atsushi Hiromoto, Kenji Suzuki, Yoshiyuki Watanabe, Yosuke Ishii

**Affiliations:** 1 Department of Cardiovascular Surgery, Nippon Medical School Musashikosugi Hospital, Kawasaki, Kanagawa, Japan; 2 Department of Cardiovascular Surgery, Nippon Medical School, Tokyo, Japan

**Keywords:** lower extremity peripheral arterial disease (PAD), extensive endarterectomy, inguinal-lifting

## Abstract

This case report presents a case of lower extremity peripheral arterial disease (PAD) with severe calcified lesions extending from the external iliac artery to the bifurcation of the superficial femoral artery, involving the groin region. Owing to the complexity and risk of complications, such as infections and delayed wound healing, revascularization was performed with extensive endarterectomy. The inguinal-lifting technique (ILT) was used to provide safe surgical access and minimize complications. The procedure resulted in successful revascularization with no major wound-related issues. The ILT proved to be an effective approach for safe, extensive endarterectomy in complex PAD cases.

## Introduction

Critical limb ischemia with long-segment occlusive disease affects the iliofemoral artery and often requires extensive endarterectomy extending across the inguinal ligament. Although this approach can achieve effective revascularization, a transverse incision extending across the groin is sometimes required to obtain an adequate surgical field. However, this type of skin incision is frequently associated with wound complications, particularly lymphatic leakage, infection, or hematoma formation, which remain among the most common postoperative morbidities. Reported rates of such complications following common femoral artery (CFA) endarterectomy range from 13% to 47%.^1,2)^

The inguinal-lifting technique (ILT) was adopted to minimize these wound-related complications while maintaining sufficient surgical exposure. This method lifts the inguinal region without dividing the inguinal ligament, thereby preserving anatomical integrity and reducing the risk of groin-related complications.

This report presents 5 cases of successful iliofemoral revascularization using extensive endarterectomy in combination with the ILT.

## Case Report

In total, 5 patients (4 men) were treated (mean age, 78 ± 5.7 years) were treated for chronic limb-threatening ischemia (CLTI) caused by long-segment arterial occlusion extending from the external iliac artery (EIA) to the femoral artery, including the posterior aspect of the inguinal ligament. One patient underwent emergency surgery. **Table 1** shows a summary of clinical characteristics and procedures.

**Table 1:** Clinical summary of 5 cases undergoing the inguinal-lifting technique

Case	Age (years)	Sex	Indication	Emergency	Inflow procedure	Outflow procedure	Patch material	Operative time (min)
1	83	F	CLTI	No	None	None	Bovine	209
2	85	M	CLTI	No	Crossover EIA–EIA	None	Vein	204
3	81	F	CLTI	Yes	Crossover EIA–EIA	AK–FP bypass	Bovine	533
4	71	M	CLTI	No	Crossover EIA–EIA	AK–FP bypass	Bovine	301
5	72	M	CLTI	No	Crossover EIA–EIA	BK–FP bypass	Bovine	427

M: male; F: female; CLTI: chronic limb-threatening ischemia; EIA: external iliac artery; AK: above knee; FP: femoropopliteal; BK: below knee

In all cases, the EIA was exposed via a retroperitoneal approach through an oblique incision above the inguinal ligament on the affected side. A separate longitudinal incision was made below the inguinal ligament to expose the CFA, superficial femoral artery (SFA), and deep femoral artery (DFA). The groin was lifted using retractors or gauze placed through the upper and lower incisions, enabling extensive endarterectomy from the distal EIA to the DFA, including the posterior inguinal region, followed by distal intimal fixation. Moreover, 4 of 5 patients underwent an EIA-to-EIA crossover bypass as the inflow procedure. Two new patients underwent femoral–above-knee popliteal artery bypass, and 1 underwent bilateral femoral–below-knee popliteal artery bypass as the outflow procedures. All grafts were constructed using 6-mm polytetrafluoroethylene. Patch angioplasty was performed in all cases using an autologous vein in 1 case and the bovine pericardium in the remaining 4.

**Fig. 1:**
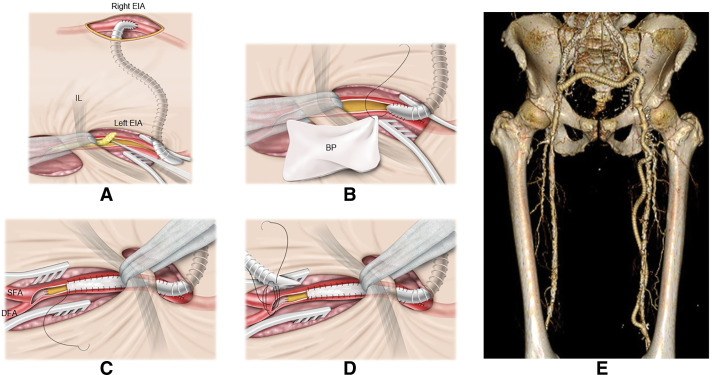
(**A**) For the central anastomosis of the inflow procedure, a PTFE graft is anastomosed to the external iliac artery on the unaffected side and subsequently tunneled beneath the rectus abdominis muscle to the contralateral side. Endarterectomy is then performed for the occlusive arterial lesion extending from the external iliac artery to the femoral artery, with retraction of the inguinal ligament. (**B**, **C**) Following endarterectomy, the PTFE graft is anastomosed to the external iliac artery as the distal anastomosis of the inflow procedure. From this site, a bovine pericardial patch is sutured beyond the inguinal ligament. At the distal portion of the common femoral artery, the intimal flap is sutured and fixed. (**D**) Patch angioplasty is completed so as to preserve the proximal anastomotic site for the femoropopliteal bypass, and a PTFE graft is anastomosed at this site. (**E**) Postoperative 3-dimensional computed tomography of case 4. EIA: external iliac artery; IL: inguinal ligament; BP: bovine pericardium; SFA: superficial femoral artery; DFA: deep femoral artery; PTFE: polytetrafluoroethylene

The surgical procedure of case 4 is illustrated as a representative example. The patient presented with CLTI of the left lower extremity. A femorofemoral bypass was constructed using the right EIA as the inflow source, followed by a femoropopliteal (FP) bypass. During the procedure, endarterectomy of the iliofemoral segment was performed using the ILT, which was similarly employed in all 5 cases (**Fig. 1**).

The mean operative time was 335 ± 127 min. Although no perioperative complications occurred, restenosis developed at the patch site in case 1, 11 months following isolated endarterectomy, and an EIA–CFA bypass was subsequently performed. Consequently, limb salvage was achieved in all patients. During follow-up (27 ± 14 months), no wound-related complications associated with the ILT were observed. In addition, no aneurysmal formation occurred at the endarterectomy site.

## Discussion

In cases where long occlusive lesions extended from the EIA to the CFA, an initial endovascular procedure was undertaken to secure arterial inflow. Subsequently, endarterectomy of the CFA and the distal EIA was performed. Adequate exposure was achieved by incising the inguinal ligament and retracting it superiorly. This hybrid approach is considered a standard strategy for managing complex iliofemoral occlusive disease.^3)^ However, in anatomical conditions such as long-segment occlusions or heavily calcified lesions where endovascular therapy may not provide durable results, a crossover bypass using the contralateral EIA as the inflow source is considered to offer better long-term outcomes.^4)^ When distal lesions were present in such cases, surgical intervention both above and below the inguinal ligament was required.

The ILT was developed to address the difficulties often encountered during procedures involving the inguinal ligament. Limited surgical visibility and complications arising from manipulation of this region often require a vertical incision that crosses the inguinal ligament; however, this incision can increase the incidence of postoperative complications, such as lymphatic leakage and inguinal hernia, which are well documented in cases of femoral artery exposure. For these cases, an approach that preserves the inguinal ligament while maintaining a wide and stable operative field was developed.

In our current practice, we use gauze traction to lift the inguinal region. Compared with conventional retractors, which often fail to provide sufficient stability in the groin area, gauze traction improves lateral mobility and flexibility in adjusting the surgical view. This method has been proven useful in navigating anatomical variations and securing adequate exposure.

However, this technique is limited by its dependence on an assistant to maintain traction. Although this is manageable when residents or students are present, our department often operates under limited staff conditions. Thus, the use of a mechanical solution, such as a retractor system that can facilitate stable, hands-free elevation, would be highly desirable.

In this series of cases, an inflow procedure was added at the endarterectomy site in 4 patients, and 3 of these cases underwent FP bypass as the outflow procedure. In case 1, in which the SFA was occluded and only patch angioplasty of the DFA was performed, thrombosis developed within the CFA. However, after performing FP bypass during reoperation, no further thrombosis occurred. These findings indicate that endarterectomy performed through inguinal lifting is beneficial only when adequate outflow is ensured.

Some researchers suggest that eversion endarterectomy could circumvent the need to manipulate the inguinal ligament. This method involves everting the arterial wall, performing endarterectomy without introducing foreign materials, and closing the artery primarily and is a viable option in selected cases.^5)^ However, the method does not universally guarantee vascular stability, particularly in patients with severe arterial sclerosis or residual intraluminal damage. These conditions may predispose patients to restenosis or even arterial rupture following primary closure.^3)^

Conversely, ILT allows for direct visualization and stable handling of the vessels while preserving critical structures. Based on our experience, this technique provides a favorable balance between exposure and safety. Although the technique is currently performed manually, future refinements should focus on creating a device that can reliably lift the inguinal region without continuous manual assistance.

## Conclusion

Thus far, endarterectomy of the iliofemoral lesion, serving as both the inflow and outflow source for bypass surgery, may yield favorable outcomes. In addition, the ILT avoids the vertical incision across the inguinal ligament—often linked to higher complication rates—while still securing an adequate operative field, making it a useful and effective approach.

### Additional Remarks

This study was presented at the 55th Annual Meeting of the Japanese Society for Cardiovascular Surgery (Yamaguchi, Japan, March 2025).
